# Chemokine ligand 2: beyond chemotaxis—a multifaceted role in tumor progression

**DOI:** 10.3389/fimmu.2025.1685474

**Published:** 2025-11-18

**Authors:** Yeying Jin, Yixuan Wang, Rui Yang

**Affiliations:** 1 Department of Breast Surgery, Shanxi Cancer Hospital/Chinese Academy of Medical Sciences Cancer Hospital Shanxi Hospital/Affiliated Cancer Hospital of Shanxi Medical University, Taiyuan, China; 2 School of Basic Medical Sciences, Shanxi Medical University, Jinzhong, China

**Keywords:** chemokine ligand 2, tumor microenvironment, immune oncology, immune cells, tumor metastasis, targeted therapy

## Abstract

Chemokine ligand 2 (CCL2) is a key regulatory molecule in the tumor microenvironment (TME) participating in the occurrence, progression, and metastasis of tumors through complex mechanisms. This paper systematically reviews the production and regulation of CCL2 in tumors and its pleiotropic effects. CCL2 can be continuously produced by tumor cells, stromal cells, and host–tumor interactions through constitutive secretion, microenvironmental stimulation response, and interaction network. Its expression is regulated by transcription factors such as Nuclear factor-kappa B (NF-κB), signal transducer and activator of transcription 3 (STAT3), and activator protein 1 (AP-1); single nucleotide polymorphisms (SNPs); and epigenetic modifications such as DNA methylation and noncoding RNA. Inflammatory factors (such as tumor necrosis factor [TNF]-α, interleukin [IL]-1β, and IL-6) and hypoxia signal in the TME further amplify CCL2 secretion through the activation of NF-κB, MAPK, and other pathways, forming a positive feedback loop. CCL2 directly promotes the proliferation, migration, and epithelial–mesenchymal transition of cancer cells by activating CCR2 receptor and its downstream PI3K/AKT, MAPK, and other signaling pathways and remodels the immunosuppressive microenvironment by recruiting tumor-associated macrophages and myeloid-derived suppressor cells. Furthermore, CCL2 drives tumor invasion and distant metastasis by inducing angiogenesis, enhancing matrix metalloproteinase activity, and promoting premetastatic niche formation. Although clinical trials targeting the CCL2–CCR2 axis have been carried out, the efficacy is limited by the redundancy of CCL2 expression and its crosstalk with other factors. Given our incomplete understanding of its mechanism, the development of combined strategies or miRNA, epigenetic intervention, and other source regulation methods is necessary. This study provides a theoretical basis for understanding the tumor regulatory network of CCL2 and the development of precise targeted therapy.

## 1 Introduction

The occurrence and progression of cancer are far from a simple cell-autonomous event, but a complex ecosystem driven by gene mutation, microenvironmental remodeling, and immune evasion. From the initial accumulation of genetic abnormalities to malignant transformation, tumor cells gradually get rid of growth regulation, recruit and reprogram surrounding stromal cells by secreting a variety of cytokines, thereby jointly constructing a tumor microenvironment (TME) that supports their survival and spread (1). This dynamic network includes immune cells, fibroblasts, endothelial cells and extracellular matrix (ECM), especially tumor-associated macrophages (TAM) (2). The interaction of cytokines, chemokines and growth factors promotes the growth of vascular tissues and the metastasis of tumor cells. It also prompts the host immune response to gradually change from the initial immune attack to immune evasion, distorting the local inflammatory response into a cancer-promoting engine. In addition, cytokines secreted by the primary tumor also lay the foundation for the formation of the premetastatic niche by “preconditioning” the organs that will be metastasized in the future (3).

Among the many molecules regulating TME, chemokines have become a key bridge connecting inflammation and tumor progression due to their core functions of mediating immune cell migration and activation (4). Among them, chemokine ligand 2 (CCL2) has attracted much attention owing to its pleiotropic and contradictory functions. CCL2, also known as monocyte chemoattractant protein-1 (MCP-1), was first isolated and purified from the culture supernatant of peripheral blood monocytes and tumor cell lines in 1989 (5). Early studies have emphasized its anti-tumor effect by recruiting monocytes/macrophages (6). In the study of non-intracranial tumors, CCL2 gene transduction has been regarded as an effective vaccine strategy to enhance the host cell defense ability against tumor cell attack (7). Furthermore, studies have shown that CCL2 synergistically with lipopolysaccharide (LPS) can activate the tumoricidal properties of macrophages, thereby reducing lung metastasis of colon cancer (8). However, with a deeper understanding of the complexity of TME, studies have found that the pro-tumor properties of CCL2 far outweigh the anti-tumor effects (9). On the one hand, its abnormally high expression attracts immunosuppressive cells such as CCR2^+^ TAMs through chemotaxis, activates pro-cancer pathways such as PI3K/AKT and NF-κB, and induces EMT, directly promoting tumor invasion and metastasis[ (10). On the other hand, CCL2 is closely related to angiogenesis, matrix degradation and the up-regulation of immune checkpoint molecules, and forms a positive feedback loop with inflammatory factors such as TNF-α to further consolidate the immunosuppressive microenvironment (11, 12). This transformation from “guardian” to “traitor” highlights the hijacking of the host physiological mechanism by tumors, and also makes CCL2 one of the core molecules to understand the malignant progression of tumors.

As cytokines involved in cancer progression, neutralizing inhibitors targeting IL-1, IL-4, TGFβ, and IL-10 have shown the potential to improve cancer (13). However, due to the pleiotropic, dual-action nature of cytokines in cancer and the redundant mechanisms of multicellular receptors, the results of relevant clinical trials are often not ideal, and many trials are abandoned (14). Blocking the CCL2-CCR2 pathway in cancer has also been investigated in a variety of clinical trials. Some trials have reported positive responses, but the efficacy of anti-CCL2 antibodies or CCR2 antagonists as monotherapies is not ideal (15). Therefore, understanding the production mechanism and downstream effects of CCL2 in TME is not only the key to reveal the logic of tumor progression, but also provides a theoretical fulcrum for the development of CCL2 therapeutic strategies.

**Figure 1:**
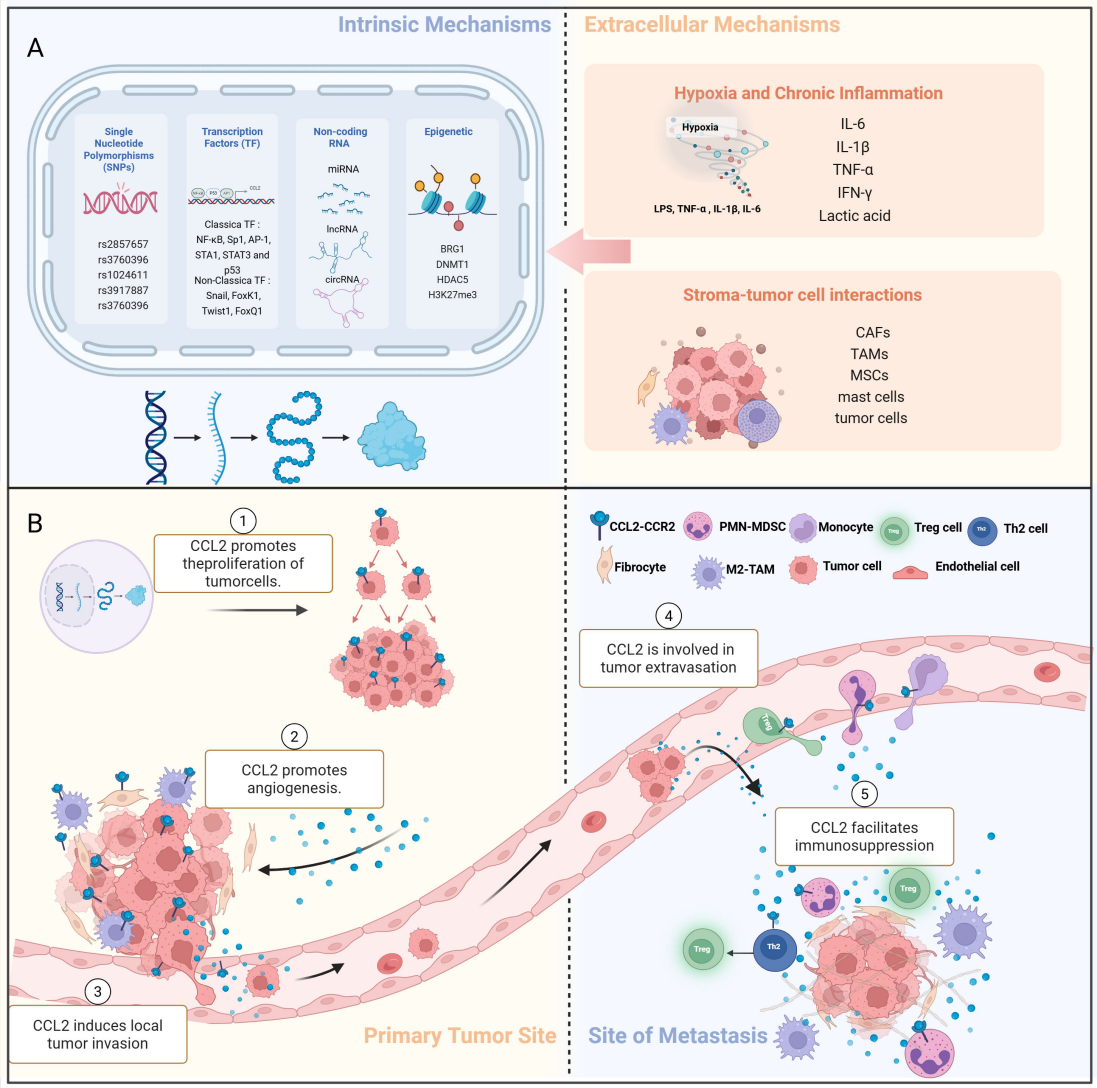
Production and pleiotropic regulation of CCL2 in the TME. **(A)** (1) The intrinsic cellular mechanism of CCL2 production:single nucleotide polymorphisms (SNPs), epigenetic regulation, non-coding RNA regulation. (2) The extrinsic cellular mechanism of CCL2 production:hypoxia and chronic inflammation; stromal tumor-cell interactions. **(B)** (1) Regulation of CCL2 production in TME. (2) CCL2 promotes the proliferation of tumor cells, enhancing their growth and survival. (3) CCL2 directly or indirectly promotes angiogenesis, the formation of new blood vessels, which supports tumor growth. (4) CCL2 induces local tumor invasion, allowing cancer cells to spread beyond the primary tumor site. (5) CCL2 is involved in the process of tumor extravasation. (6) CCL2 facilitates immunosuppression, inhibiting the immune response against cancer cells, thereby promoting tumor growth and metastasis. M2-TAM, M2 tumor-associated macrophages;Treg cell, Regulatory T cells;Th2 cell, Helper T cells; PMN-MDSC, Polymorphonuclear myeloid-derived suppressor cells; SNP, single nucleotide polymorphism.

This article systematically reviews the biological characteristics of CCL2-CCR2, as well as the constitutive secretion of CCL2 in the TME, its microenvironment-responsive expression, and the multi-dimensional regulation resulting from tumor-stroma interactions. It focuses on the pleiotropic roles of CCL2 in the EMT, angiogenesis, immune escape, and metastatic cascades. Finally, we discuss the limitations and directions of therapeutic interventions targeting CCL2. The aim is to provide a theoretical basis for precise anti-tumor strategies targeting CCL2. Please refer to **Figure 1** for further details. Created in https://BioRender.com.

## 2 Biological characteristics and functions of CCL2

CCL2, also known as MCP-1, is one of the earliest discovered human CC chemokines (16). It is a 76-amino acid basic protein encoded by the *CCL2* gene located on chromosome 17q11.2-q12 (16).In the TME, monocytes and macrophages serve as the primary producers of this molecule (17). As a small-molecule cytokine, CCL2 shows remarkable pro-inflammatory chemotactic activity. Under normal physiological conditions, CCL2 recruits immune cells such as monocytes, macrophages, memory T cells, natural killer (NK) cells, and dendritic cells from the blood to the inflammation site through the chemokine gradient, which helps maintain the normal distribution and function of immune cells (18). In addition to its chemotactic function, CCL2 has a multifaceted role in the regulation of immune cell function. In the inflammatory response, CCL2 can enhance the adhesion of monocytes to vascular endothelium by increasing the expression of integrin β2 family and the release of arachidonic acid, thereby strengthening the retention capacity of monocytes (19, 20). It also regulates the secretion of effector molecules and the autophagy, killing, and survival of immune cells (21). Moreover, CCL2 participates in the development, differentiation, maturation, homing, and interaction of immune cells (18, 22, 23); initiates and maintains inflammatory responses; and balances the intensity of inflammation by regulating the polarization state of macrophages and cytokine secretion (24). In tissue repair, CCL2 promotes the migration and polarization of monocytes and macrophages for tissue remodeling and angiogenesis (25–27).

**Figure 2:**
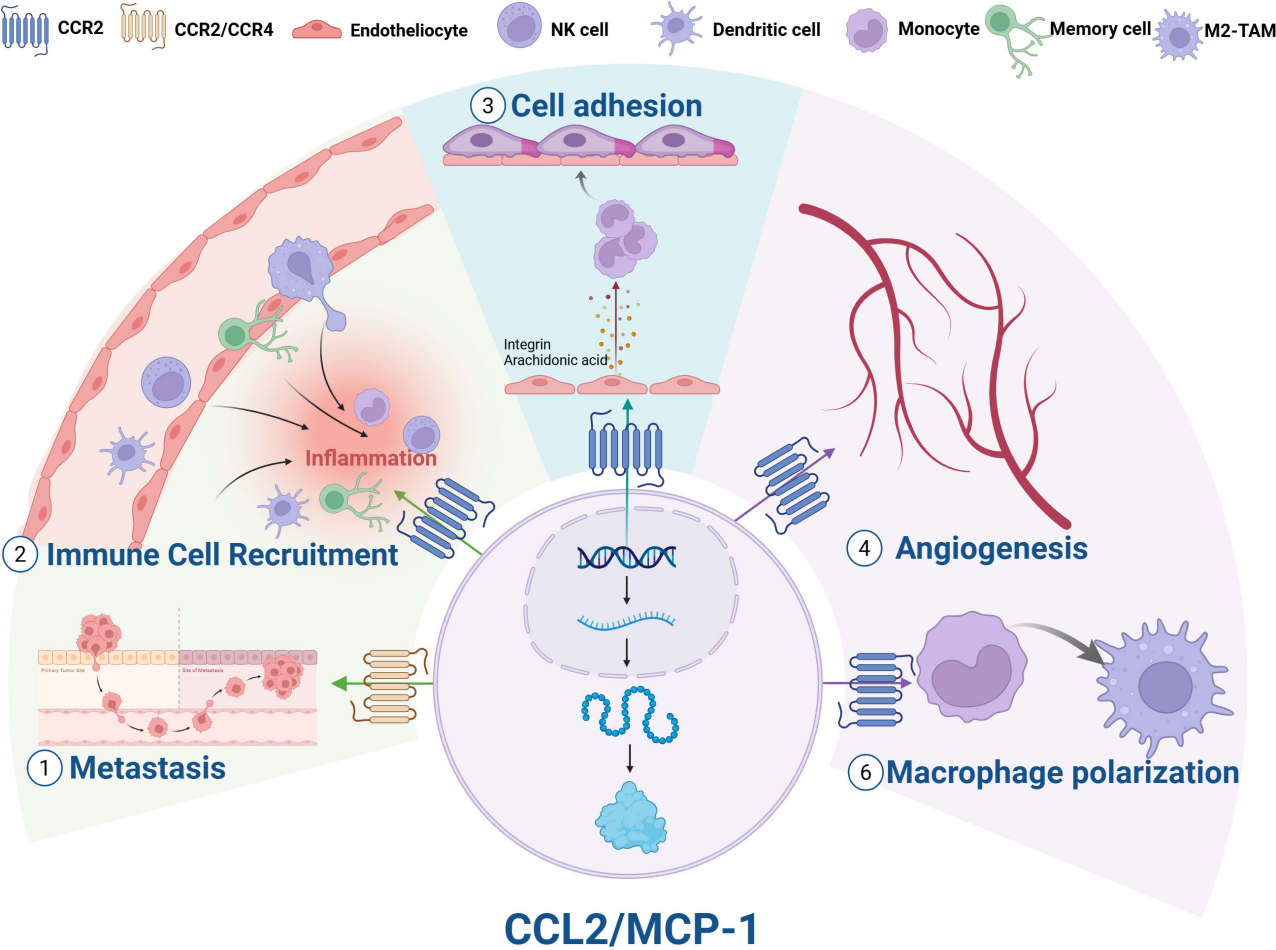
Biological functions of CCL2. (1) Metastasis; (2) Immune cell recruitment; (3) Cell adhesion; (4) Angiogenesis; (5) Macrophage polarization; (6) Enhanced tumor growth and metastasis. NK cell, natural killer cell; M2-TAMs, M2 tumor-associated macrophages.

The main receptor of CCL2 is C-C chemokine receptor type 2 (CCR2), a seven-transmembrane G protein-coupled receptor with extremely strong affinity (*K_d_* = 0.77 nM) (28, 29). In terms of binding mechanism, the N-terminus of CCL2 forms a key hydrogen bond with the third transmembrane domain (TM3) and the second extracellular loop (ECL2) of CCR2. These interactions make CCL2 the most potent ligand to activate CCR2 signaling (30). The binding of CCL2 and CCR2 is essential for initiating signal transduction pathways and stimulating cell migration. Upon binding to CCR2, CCL2 activates the G protein-coupled receptor signaling pathway to initiate an intracellular signaling cascade, which includes PI3K/AKT, mitogen-activated protein kinase (MAPK)/p38, protein kinase C (PKC), calmodulin-dependent protein kinase II, and JAK/STAT3 pathways (31). By activating these signaling pathways, CCL2 regulates cytoskeleton reorganization and phospholipase C-dependent calcium release, which plays a key role in anti-apoptosis, angiogenesis, and cell invasion and migration. CCL2 can also bind to other G protein-coupled receptors. For example, the CCL2–CCR4 signaling axis is a key mechanism to promote brain metastasis in breast cancer (32) and mediates cell migration in head and neck squamous cell carcinoma (33). Furthermore, CCL2 can bind to atypical receptors (ACKR1 and ACKR2). Although they have a sevenfold transmembrane structure similar to classical chemokine receptors, atypical receptors are not involved in G-protein-mediated signal transduction and do not affect intracellular calcium concentration. They also do not trigger cell migration (34, 35). Although they do not generate signals through G proteins, they are involved in the formation of chemokine gradients and regulate chemokine bioavailability and signaling by internalizing and degrading or redirecting CCL2, acting as regulatory components of the chemokine network (36, 37). The biological function of CCL2 is shown in **Figure 2**.

## 3 Mechanisms of CCL2 production in the tumor microenvironment

**Figure 3:**
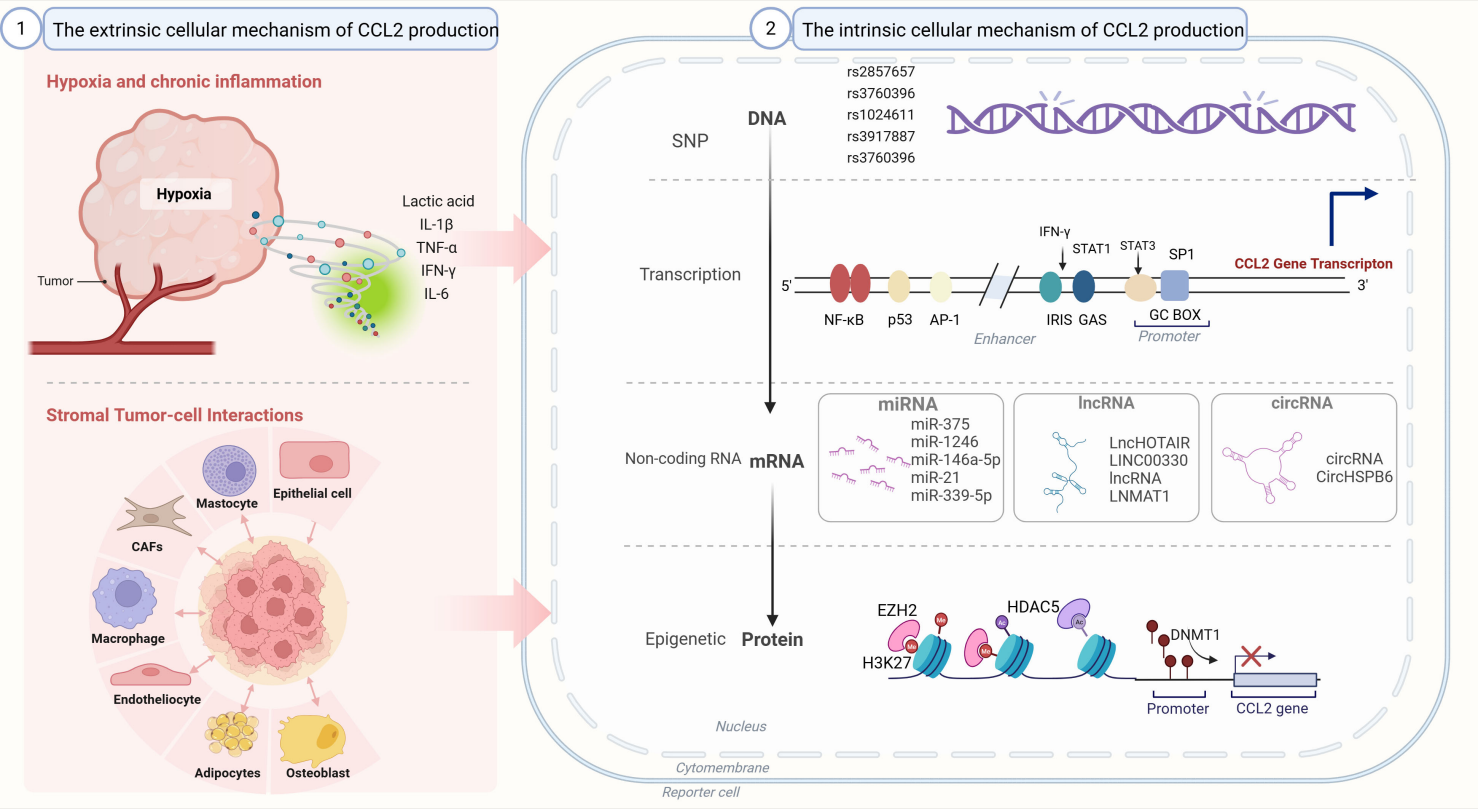
Regulation of CCL2 production. The intrinsic cellular mechanism of CCL2 production: single nucleotide polymorphisms (SNPs), epigenetic regulation, non-coding RNA regulation. (2) The extrinsic cellular mechanism of CCL2 production:hypoxia and chronic inflammation; stromal tumor-cell interactions. SNP: single nucleotide polymorphism. CAFs, Cancer-associated fibroblasts; miRNA, MicroRNA; lncRNA, Long non-coding RNA; circRNA, Circular RNA; SNP, Single nucleotide polymorphism; NF-κB, Nuclear factor kappa-light-chain-enhancer of activated B cells; p53, Tumor protein p53; AP-1, Activator protein 1; IRIS, Inflammation and response sensor; GAS, Gamma interferon activation site; STAT1, Signal transducer and activator of transcription 1; STAT3, Signal transducer and activator of transcription 3; GC BOX, Guanine cytosine box; EZH2, Enhancer of zeste homolog 2; HDACs, Histone deacetylases; H3K27, Histone H3 lysine 27; DNMT1, DNA methyltransferase 1.

In the TME, CCL2 is synthesized by various cell types such as cancer cells and stromal cells in response to various stimuli. The production pathways of CCL2 can be summarized into the following three categories: (1) Cells constitutively produce CCL2; (2) The responsive expression of CCL2 to the stimulatory factors in TME; (3) Stromal tumor-cell interactions produce CCL2. These three mechanisms interact to construct a complex regulatory network, which leads to the high level of CCL2 expression in the TME (**Figure 3**), and also provides a large number of precise targets for CCL2-targeted therapy.

### 3.1 Cells constitutively produce CCL2

Several studies have confirmed that overexpression of various transcription factors in cancer cells can affect the transcription process of CCL2. Tumor cells can continuously and autonomously secrete CCL2 through intrinsic mechanisms due to intrinsic gene or mutation or epigenetic changes without external stimulation.

#### 2.1.1 Single nucleotide polymorphisms (SNPs) regulate CCL2 expression

Single nucleotide polymorphisms (SNPs) in the regulatory regions of cytokine genes may affect CCL2 transcription and alter the expression levels of these genes in specific populations (38). Among CCL2 SNPs, +764 C/G (rs2857657), −927 G/C (rs3760396), −2518 A/G, and −2835C/A are associated with high CCL2 expression; −2518 A/G (rs1024611), −927 G/C (rs3760396), and −2835 C/A (rs2857654) are located in the promoter regulatory region; and +764 C/G and CCL2 14bp I/D (rs3917887) are located in the intronic region (39, 40). Meanwhile, −2518A/G (rs1024611) is the most widely studied CCL2 regulatory SNP and has the most well-defined function in tumors; its G allele rs1024611G is associated with high CCL2 expression and poor clinical prognosis in a variety of cancers (41). By contrast, rs1024611A is associated with low CCL2 expression (42). rs2857656 (−362 G/C), rs1024611(−2518 A/G), and rs4586 (Cys35Cys) of CCL2/MCP1 are positively associated with elevated circulating levels of CCL2 and significantly associated with an increased risk of breast cancer (43). CCL2 rs1024611 SNP may also be associated with the occurrence and development of cervical cancer, deep stromal invasion, large tumor diameter, and parametrial invasion (44). The effects of other SNPs on CCL2 expression and their diagnostic values in different cancers are detailed in **Table 1**.

**Table 1:** SNPs associated with different cancer types in CCL2.

Forms of genetic variation	Region	Genetic models	Expression of CCL2	Cancer types	Effects on cancer
-2518A/G (rs1024611)		AA	Down	Gynecologic cancer (breast cancer, etc.)	The AA genotype is a protective factor for gynecologic cancer in Asians, but AG is a risk factor in Europeans (45).
	promoter regulatory region	GA + GG	Up	Gastric cancer (GC) Nasopharyngeal cancer (NPC) Colorectal cancer (CRC) and colorectal liver metastases (CRLM)	The GA + GG genotype is associated with a higher risk of GC and NPC in the Chinese population and is associated with advanced GC (46, 47). It is positively associated with increased risk of CRC and CRLM, and with poor prognostic features of CRC (48).
		GG	Up	Breast cancer (BC), endometrial cancer (EC), cervical cancer (CC), ovarian cancer (OC), renal cell carcinoma (RCC) Philadelphia chromosome negative myeloproliferative neoplasm (MPN) melanoma (MM)	Among gynecologic cancers in Asians, carriers of the GG genotype have a higher risk than carriers of the AA or AG genotypes (45), and associated with cancer susceptibility in Caucasians (49). The GG genotype is more common in patients after PMF and PV/ET-MF and is significantly associated with fibrosis grade (50). It is associated with melanoma progression in Caucasians (51).
–927 G/C (rs3760396)	promoter regulatory region	GC + GG	Up	Adenosquamous Lung Cancer (ASC)	Is significantly associated with an increased risk of adenosquamous lung cancer (52).
−2835 C/A (rs2857654), −1811 A/G (rs3760399), +3726 T/C (rs2530797)	promoter regulatory region	AA, AG+GG, TT	Up	Prostate cancer (PCa)	−2835 C/A (AA), −1811 A/G (AG+GG), +3726 T/C (TT) are associated with higher aggressiveness of PCa (40).
CCL2 14bp I/D (rs3917887)	intronic region	ID, DD	–	Esophageal cancer (EC)	Is associated with a high risk of EC (53).
		ID + DD	–	Prostate (PCa) Bladder (BC)	Is associated with high risk of PCa and BC (54, 55).
CCL2I/D, CCL2A2518G	intronic region Promoter regulatoryregion	D-A	–	Bladder cancer (BC)	Is associated with high risk of BC in north Indian population (55).

PV, Polycythemia vera; ET, Essential thrombocythemia; MF, Myelofibrosis.

#### 2.1.2 Epigenetic modifications regulate CCL2 expression

Epigenetic alteration refers to the process of regulating gene expression through chemical modification or chromatin structure changes without altering the DNA sequence. CCL2 expression is affected by a variety of epigenetic regulatory mechanisms, including DNA methylation, histone modification, chromatin remodeling.

Posttranslational modifications of histone N-terminal tails, including phosphorylation, acetylation, ubiquitination, and methylation, can regulate nucleosome structure (56, 57). Among them, acetylation usually activates gene expression by promoting the interaction between transcription complexes and DNA (57). Methylation is closely related to the activation or repression of genes (58). In small cell lung cancer, DNA methyltransferase 1 (DNMT1) inhibits CCL2 expression by adding methyl groups to CpG islands in the promoter region of *CCL2* gene, preventing TF binding (59). As a histone methyltransferase, Enhancer of Zeste Homolog 2 (Enhancer of Zeste Homolog 2) catalyzes the trimethylation of histone Histone H3 Lysine 27 (H3K27) and represses gene expression. Its binding to the enhancer region of CCL2 leads to an increase in the trimethylation level of H3K27 and ultimately inhibits the transcription of CCL2 (59). In breast cancer, EZH2 and DNMT1 act synergistically to regulate CCL2 expression. EZH2 can recruit DNMT1 to the promoter region of CCL2 gene, leading to DNA hypermethylation, and then up-regulate miR-124-3p to further inhibit CCL2 expression (60). miR-124-3p regulates CCL2 expression at the posttranscriptional level by targeting the 3′ untranslated region (3′ UTR) of CCL2 and degrading its mRNA or inhibiting its translation (61). In macrophage inflammatory activation, transcription repressors G protein pathway suppressor 2 (GPS2) and silencing mediator for retinoid and thyroid hormone receptors (SMRT) regulate enhancer and silencer remodeling via the transcription of long noncoding enhancer RNAs, consequently suppressing CCL2 transcription (62). In an iKPC mouse model, histone deacetylase 5 (HDAC5) was found to be a potential downstream target of the proto-oncogene KRAS. Its inhibition can lead to the down-regulation of negative regulator Socs3, which in turn increases CCL2 expression and promotes the infiltration of macrophages recruited by CCL2 (63). Brahma-related gene 1 (BRG1) promotes the recruitment of acetylated histone H3 and H4 and nuclear factor NF-κB to the CCL2 promoter region by regulating chromatin structure and TF binding. This process consequently up-regulates CCL2 (64).

#### 2.1.3 Noncoding RNAs regulate CCL2 expression

Noncoding RNA Regulation (ncRNA) play a key role in the development and course of many diseases, especially cancer. The family of ncRNAs encompasses various molecular species, including microRNAs (miRNAs), long non-coding RNAs (lncRNAs), and circular RNAs (circRNAs), among other type (65). *In vitro*, long noncoding RNA H19 (H19-IRP) promotes glioblastoma multiforme (GBM) immunosuppression by binding to CCL2 and galectin-9 promoters to activate its transcription and recruiting myeloid-derived suppressor cells (MDSCs) and TAMs to induce T cell exhaustion and an immunosuppressive GBM-TME. Traditional GBM vaccines such as Rindopepimut targeting EGFRvIII and dendritic cell (DC) vaccine ICT-107 rely on T cell activation, but have limited improvement on immunosuppressed GBM-TME. However, the circular RNA vaccine against human H19-IRP activates anti-tumor T cells and modifies GBM–TME to enhance the antitumor effect (66). In the TME, miRNAs also regulate CCL2 and thus have become effective prognostic markers (67). Compared with lentivirus or protein-based gene therapy, ncRNAs have more potential in clinical application because of their simple structure and lower immunogenicity and toxicity (68). Therefore, CCL2-regulated ncRNAs serve as novel cancer biomarker and therapeutic target for cancer patients. Other ncRNAs that regulate CCL2 expression are listed in **Table 2**.

**Table 2:** Noncoding RNAs regulate CCL2 expression.

Cancer type	miRNA	Expression of CCL2	Mechanism Description	Downstream
Hepatocellular carcinoma (HCC)	miR-122 hsa_circ_0110102/miR-580-5p	Down	miR-122 targeting the 3'-UTR to inhibit CCL2 expression miR-580-5p/PPARα/CCL2 pathway reduced CCL2 secretion.	CCL2 is upregulated in the liver of miR-122 null mice, recruiting CCR2^+^CD11b^+^ Gr1^+^ immune cells to the liver (69). The secretion of CCL2 is reduced, which inhibits the release of proinflammatory cytokines by TAM through the COX-2/PGE2 pathway (70).
	miR-206	Up	Enhanced CCL2 expression through KLF4-NF-κB axis.	It promotes the recruitment of CTLs in the liver (71).
Pancreatic ductal adenocarcinoma (PDAC)	miR-155-5p	Up	——	Tregs are recruited to form an immunosuppressive tumor microenvironment, which further promotes cancer cell proliferation and vascularization (72)
Lung cancer (HC)	miR-1 and miR-206 were down-regulated, while miR-31 was up-regulated	Down	Inhibition of CCL2 expression by binding to its 3'-UTR.	miRNA mediates FOXO3a/VEGF/CCL2 signaling to promote tumor growth, angiogenesis, TAMs accumulation and lung metastasis (73).
	miR-196a	Up	It promoted CCL2 secretion through SOCS6/STAT3 pathway.	CAFs up-regulate CCL2 to promote cell migration and invasion (74).
Colorectal Cancer (CRC)	miR-506-3p	Down	It indirectly promoted CCL2 expression by regulating FoxQ1.	Up-regulation of CCL2 promotes macrophage recruitment (75).
	LncHOTAIR	Up	It directly activates downstream CCL2 in the corpus cavernosum miR-206.	Activation of CCL2 promotes the proliferation, migration and invasion of cancer cells (76).
A variety of tumors	miR-210-3p	Down	It silences CCL2 mRNA by targeting its 3'-UTR.	Hypoxia-induced miR-210-3p reduces monocyte infiltration by inhibiting CCL2 expression (77).
Bladder cancer (BLCA)	miR-1-3p	Down	It silences CCL2 mRNA by targeting its 3'-UTR.	Targeting CCL2 inhibits the proliferation and invasion of bladder cancer cells (78).
	lncRNA LNMAT1	Up	Recruitment of hnRNPL to the CCL2 promoter region promotes H3K4 trimethylation and enhances CCL2 transcription.	TAM is recruited and VEGF-C is secreted to promote lymphatic metastasis (79).
Hemangioma (HA)	CTBP1-AS2/miR-335-5p	Up	It reduces the inhibition of CCL2 by miR-335-5p through ceRNA mechanism.	miR-335-5p directly targets CCL2 and can also act together with CTBP1-AS2 of ceRNA, thereby upregulating CCL2 and promoting HA cell proliferation, migration, invasion and angiogenesis, while inhibiting apoptosis (80).
A variety of tumors	miR-375	Up	FOXK1 was regulated by targeting PPP2R2B to promote CCL2 expression.	It increase the recruitment of macrophages (81).
Breast Cancer (BC)	Exosomal miR-155	Up	It promotes CCL2 secretion by targeting SOCS6/STAT3 pathway	It upregulates CCL2 in tumor-adipocyte co-culture, which in turn recruits and polarizes M2 TAM to promote BC growth (82).
	miR-124-3p, miR-126/miR-126*	Down	miR-124-3p inhibited CCL2 expression by binding to its 3'-UTR. miR-126/miR-126* indirectly inhibited CCL2 expression by targeting SDF-1α.	EZH2 depletion leads to the stagnation of M2 TAM polarization (60. Down regulation of CCL2 expression by miR-126 and miR-126* inhibits the recruitment of mesenchymal stem cells and inflammatory monocytes to inhibit breast cancer metastasis (67).
Kaposi's sarcoma (KS)	miR-K10a	Down	Down-regulation of CCL2 expression by targeting TWEAKR.	Protects the tumor from apoptosis while inhibiting the proinflammatory response (83).
Lung adenocarcinoma (LUAD)	circRNA CircHSPB6	Up	The inhibition of let-7a-2-3p on CCL2 was relieved by sponge adsorption.	It promotes the M2 polarization and infiltration of TAMs by CCL2, and then promotes tumor cell proliferation, migration, invasion and EMT (84).
Non-small cell lung cancer (NSCLC)	miR-1246	Up	It promotes CCL2 transcription through the NF-κB signaling pathway.	Induction of proinflammatory responses in MSCS/stromal cells (85).
	miR-146a-5p	Down	It silences CCL2 mRNA by targeting its 3'-UTR.	Tumor suppressor function (86).
Esophageal squamous cell carcinoma (ESCC)	LINC00330	Down	It binds to CCL2 protein to form the RNA\u2012 protein complex, thereby inhibiting translation and function.	Inhibition of CCL2/CCR2 axis and its downstream signaling pathways can inhibit ESCC, while blocking CCL2-mediated TAM polarization reprogramming in a paracrine manner (87).
Colorectal cancer (CRC)	LncHOTAIR	Up	It directly activates CCL2 downstream of miR-206 in the corpus cavernosum.	It promotes the proliferation, migration and invasion of cancer cells (76).
Oral squamous cell carcinoma (OSCC)	miR-21, miR-339-5p	Up	Activation of NF-κB promotes CCL2 transcription. miR-339-5p indirectly activated the NF-κB pathway and upregulated CCL2 expression by targeting TSPAN15.	It promotes monocyte reprogramming (88). miR-339-5p promoted OSCC metastasis by up-regulating CCL2 (89).
	miR-124	Down	It directly inhibits the expression of CCL2.	It can inhibit CCL2 production by CAFs and promote the growth and migration of OCCs (90).

PGE2, Prostaglandin e2; COX-2, Cyclooxygenase-2; CTLs, Cytotoxic T cells; Tregs, Regulatory T cells; Cancer-associated fibroblasts, CAFs; FOXQ1, Forkhead box q1; FOXO3a, Forkhead box o3a; VEGF, Vascular endothelial growth factor; SOCS6, Suppressor of cytokine signaling 6; STAT3, Signal transducer and activator of transcription 3; ceRNA, Competing endogenous RNA; 3’-UTR, 3’ Untranslated region; FOXK1, Forkhead box k1; EZH2, Enhancer of zeste homolog 2; TAM, Tumor-associated macrophages; TWEAKR, TNF-like weak Inducer of apoptosis receptor; EMT, Epithelial-mesenchymal transition.

### 3.2 Microenvironmental stimulation regulate CCL2 expression

In addition to the constitutive expression of CCL2 driven by intrinsic genetic variations in cells, the chronic inflammatory state and hypoxic conditions within the TME further promote abnormal CCL2 expression (91). It is worth noting that these factors usually do not act independently but rather precisely regulate the expression profile of chemokines in the TME through synergistic effects. For instance, in papillary thyroid carcinoma (PTC), the combined stimulation of IFN-γ and TNF-α can significantly synergistically upregulate the expression of CXCL9 and CXCL11, activating the anti-tumor immune pathway (92). Therefore, the synergy among inflammatory factors and the positive feedback network form the basis for constructing a complex regulatory network.

#### 3.2.1 Chronic inflammation regulate CCL2 expression

Cytokines including, IL-6, and IL-1β engage in positive feedback interactions with CCL2 in the TME—a phenomenon with important implications for tumor therapy.

TNF-α, a key pro-inflammatory cytokine in tumors (93), is constitutively overexpressed via its receptor on tumor cells, correlating with sustained NF-κB activation and elevated MCP-1/CCL2 expression. In breast and ovarian cancers, the TNF-α secreted by malignant cells potently induces CCL2/MCP-1 expression, with nearly a twofold increase upon TNF-α stimulation compared with its inhibition (94, 95). Moreover, the TNF-α secreted by CCL2-recruited TAMs further amplifies CCL2 production, establishing a reinforcing cycle that perpetuates CCL2 activation (93).

IL-1β, another proinflammatory cytokine belonging to the IL-1 family (96), promotes CCL2 expression in macrophages and tumor cells within the TME (97). The coordinated action of TNF-α and IL-1β leads to a sustained CCL2 release from tumor and endothelial cells (98, 99). Furthermore, IL-1β and CCL2 mutually enhance each other’s production, forming a feedback loop that amplifies macrophage infiltration into tumor tissues (100).

IL-6, a pivotal tumor-promoting cytokine, induces CCL2 secretion via STAT3 activation; the two engage in macrophage-mediated feedback amplification (101, 102). Functionally, IL-6 and CCL2 exhibit synergistic effects: certain p53 protein isoforms enhance cancer cell migration and metastasis by upregulating both cytokines (103). The compound N-2 suppresses tumor growth through its concurrent inhibition of IL-6 and CCL2 via p53 and NF-κB pathways (104). IL-6 and CCL2 induced by CMTM4 upregulate MDSC expansion in the TME, and silencing CMTM4 enhances anti-PD-1 efficacy (105). Oligonucleotide–chitosan complexes suppress IL-6 and CCL2 production and cooperate with p53 to inhibit ATM signaling and tumor progression (106). These findings underscore the therapeutic potential of targeting the IL-6/CCL2 axis to overcome resistance to immunotherapy.

#### 2.2.2 Hypoxia regulates CCL2 expression

Hypoxia is a common feature of solid tumors and a key regulator of the TME (107). Chronic (continuous, uninterrupted) and periodic (intermittent, transient) hypoxia both occur in malignant tumors. In the early stages of tumor development, the inability of blood vessels to provide oxygen to the tumor interior leads to the formation of chronic hypoxic regions (108). In the later stages of tumor development, the abnormal structure of tumor-generated blood vessels leads to periodic hypoxia in different regions inside the tumor (109). Under chronic hypoxia, oxygen-dependent Jumonji histone demethylase activity is reduced, which increases histone methylation in the *CCL2* gene promoter, thereby reducing CCL2 expression (110). In addition, hypoxia induces histone deacetylase 1 (HDAC1) activation via CK2 (111). The activated HDAC1 forms a complex with p65 NF-κB and acts as a transcriptional repressor through histone deacetylation to reduce CCL2 expression (112). HIF-1α is activated as a major TF in chronic hypoxia; it can bind to the hypoxia response element (HRE) in the promoter region of *CCL2* gene and directly promote *CCL2* transcription (113). Hypoxia can also indirectly regulate CCL2 expression. Solid tumor-associated hypoxia can up-regulate the gene expression of the regulator of G protein signaling 2 (Rgs2) in MDSCs, leading to a significant increase in CCL2 (114).

The main pathway of CCL2 production is cyclic hypoxia, where NF-κB activation is the most important mechanism to increase CCL2 expression (91). The transcriptional expression of CCL2 is induced by increasing NF-κB p65/p50 binding activity (115). Hypoxia/reoxygenation (H/R) also induces CCL2 expression in melanoma cells through the synergistic effect of TFs NF-κB and SP1; this process involves the MAPK signaling pathway (91). Cyclic hypoxia can also indirectly regulate CCL2 expression. In THP-1 monocytes, hypoxia indirectly enhances CCL2/MCP-1 expression by up-regulating the receptor for advanced glycation end products through the activation of HIF-1α and NF-κB (116).

In addition to the above common tumor environmental factors, lactate accumulation and growth factors can regulate CCL2 expression in the TME. The binding of lactate to HCAR1 could activate scaffold protein 14-3-3, which in turn activates the STAT3 signaling pathway. The activated STAT3 enhances *CCL2* transcription, resulting in increased CCL2 expression (117). In breast fibroblasts, TGF-β signaling inhibits CCL2 expression and negatively regulates TAM recruitment and tumor metastasis (118).

#### 2.2.3 Integrated role of classical transcription factors in CCL2 regulation

By conducting extensive literature review, it has been found that inflammation and hypoxia signals can eventually activate a series of classical TFs, including NF-κB, Sp1, AP-1, STAT3, and p53, which jointly regulate the basal expression of *CCL2* gene. Taking these TFs as the core, we systematically sorted out their upstream stimulating factors, downstream effects, and synergistic effects as detailed in **Table 3**.

**Table 3:** The external cellular environment influences CCL2 expression through transcription factors.

The TFs upstream of CCL2	External stimuli	Binding site	Synergy	Cancer type	Downstream
NF-κB (p65/p50)	TNF-α, IL-1β, IL-33 (119) Hypoxia/reoxygenation (H/R), estrogen (120)	Distal enhancers upstream of the promoter (bp-2612 to -2603) (121)	It directly binds to the CCL2 enhancer in response to IL-1β and TNF-α (121). It synergets with Sp1 and AP-1 to promote CCL2 expression (122).	Breast cancer (BC) (123) gastric cancer(GC) (124) ovarian cancer(OC) (125) non-small cell lung cancer (NSCLC) (126)	(1) Stimulation of the PI3K/Akt pathway leads to paclitaxel resistance (125) (2) recruit TAMs (127) (3) Attract THP-1 monocytes (128)
Sp1	LPS, TNF-α, IL-1, NF-κB, IFN-γ, Hypoxia/reoxygenation (H/R) (121, 129)	Promoter proximal GC box (-64 to -59bp)	The MCP-1 promoter can be fully activated when both NF-κB and SP1 binding sites are present (130). The binding of SP1 enhances the binding activity of NF-kB and AP1, leading to the synergistic activation of the CCL2 promoter (122).	Malignant glioma (MM) (129) Colorectal cancer (CRC) (131) Human lung adenocarcinoma (132)	Recruit macrophages (131)
AP-1 (c-Jun/c-Fos)	TNF-α	-68bp upstream of promoter (133)	The binding of NF-κB and AP-1 has a synergistic effect (134)..	Glioblastoma multiforme (GBM) (135) Prostate cancer (PCA) (136)	The AP1-CCL2-TNF-α axis promotes the reprogramming of tumor cell lineages towards an invasive phenotypic state (137)
STAT3	IL-6, IL-7, Lactic acid (117)	-147 to -138bp upstream of promoter (138)	It can act in concert with SPOP, an E3 ubiquitin ligase, and the transcription factor ETV5 (139, 140)..	Bladder cancer (BC) (140) urothelial bladder cancer (UBC) (140) colorectal cancer (CRC) (75) human hepatocellular carcinoma (HCC) (141) non-small cell lung cancer(NSCLC) (142) Oral cancer(OC) (143)	(1) Macrophage migration and metastasis mediated by mesenchymal circulating tumor cells (75) (2) Induce M2 macrophage polarization and recruit CD8+ T cells (141) (3) Recruit TAMs and suppresses cytotoxic T cells activity (144).
STAT-1α	IFN-γ	The 213 bp region proximal to the promoter (145)	SP1 and STAT-1α have a synergistic effect (145)..	Astrocytoma cells (AC)	Recruit TAM (145)
P53	TNF-α, Mutation of p53 gene	2.5kb upstream of the start site (146)	Loss of tumor suppressor function. Binding of mutp53 to NF-κB significantly enhanced eRNA and mRNA expression levels of CCL2, whereas wild-type p53 did not have this regulatory ability (147).	Ovarian high-grade serous carcinoma (OHGSC) (148) Human tumor virus (HPV) positive precancerous and malignant cells (149)	(1) Promote the infiltration of immunosuppressive myeloid cell populations (148) (2) Promote invasion and metastasis (103)
HIF1α	Hypoxia	HREs (hypoxia response elements) (113)	HIF-1 binds to the HREs binding sites in CCL2 and activates its transcription.	Breast cancer cells (BC) Cervical squamous carcinoma cells (CSCC) Hepatoma cells (HC) Lewis lung carcinoma cells (LLC) Multiple myeloma (MM) (150)	(1) Recruit CCR2+ Treg (151) (2) Recruit microglias and monocytes (152)

NF-κB, Nuclear factor kappa-light-chain-enhancer of activated B cells; IL-1β, Interleukin 1 beta; TNF-α, Tumor necrosis factor alpha; LPS, Lipopolysaccharide; GC, Glucocorticoid; CTLs, Cytotoxic T cells; Sp1, Specificity protein 1; STAT1, Signal transducer and activator of transcription 1; P53, Tumor protein P53; TAMs, Tumor-associated macrophages; HRF1, Hypoxia responsive factor 1; HIF1α, Hypoxia inducible factor 1 alpha; AP-1, Activator protein 1; ETV5, ETS variant 5; EMT, Epithelial-mesenchymal transition; HIF1α, Hypoxia inducible factor 1 alpha.

Among these TFs, NF-κB plays the most crucial role in CCL2 regulation. NF-κB is located in a distal enhancer approximately 2.5 kbp upstream of the basic promoter. Two specific binding sites can be found in the distal region of the *CCL2* gene promoter (153). The far upstream κB binding site (bp −2612 to −2603) is essential for the enhancer activity induced by cytokines such as IL-1β and TNF-α, and its mutation completely abolishes this activity (121).

A complex, bidirectional, positive-feedback regulatory relationship exists between NF-κB and CCL2. On the one hand, NF-κB is a key upstream regulatory factor for CCL2 expression; on the other hand, CCL2 can inhibit the M1 phenotype of TAMs through ZC3H12A, and this process depends on the NF-κB signaling pathway (154). The drug degreen can significantly inhibit the activation of the PI3K/Akt/NF-κB signaling pathway in GBM cells, thereby hindering the disease progression; by contrast, exogenous CCL2 addition can activate the NF-κB signaling and reverse the inhibitory effect of degreen on DBTRG cells (155). In hepatocellular carcinoma (HCC), CCL2 elevated PD-L1 expression by activating the NF-κB pathway (156).

#### 2.2.4 Non-classical transcription factors regulate CCL2 expression

In addition to the classic transcription factor pathways, CCL2 is also regulated by some non-transcription factor factors. PPARγ belongs to the ligand-activated transcription factor of the nuclear receptor superfamily. In anaplastic thyroid carcinoma (ATC) in humans, PPARγ agonists can inhibit the secretion of CCL2 in some tumor cells and simultaneously suppress the activation of NF-κB and ERK1/2 signaling pathways (157). Creb-binding protein (CBP) can interact with the transcription repressor Snail, which increases the promoter activity of CCL2 through the acetylation modification of Snail (158). Insulin-induced activation of the mammalian target of rapamycin complex 1 induces the dephosphorylation of transcription factor FOXK1, which directly binds to the promoter region of the *CCL2* gene and promotes its transcriptional activation. This process is independent of the classical NF-κB signaling pathway (159). Twist1 up-regulation in cancer cells activates the transcription of CCL2 mRNA, thus promoting CCL2 expression (160). In breast cancer, estrogen enhances Twist expression by activating ERα and PI3K/AKT/NF-κB signaling, which in turn induces CCL2 autocrine (120). Forkhead box Q1 (FoxQ1) is a major regulator of tumor metastasis. It can bind to the promoter region of Versican V1 gene and induce its expression, ultimately promoting CCL2 secretion (161). FoxQ1 also enhances CCL2 expression by activating Twist1 (162). CCAAT/enhancer binding protein β (C/EBPβ) may interact with AP-1 family members to drive CCL2 transcription (163). Fli-1 is a key member of the Ets TF family and can directly regulate the expression of *CCL2* gene by binding to its promoter. The interaction between Fli-1 and p65, a member of the NF-κB family, can enhance the transcriptional activity of CCL2 promoter (164). *KRAS* gene mutation is one of the most common mutations in human cancers, especially in metastatic cancers such as pancreatic cancer, lung cancer, and colorectal cancer (165). *KRAS* activation promotes CCL2 expression. In the mouse model of pancreatic ductal adenocarcinoma, *KRAS* indirectly promotes CCL2 expression by regulating HDAC5 and negative regulator Socs3, thereby shaping the immune cell composition in the TME (63).

### 3.2 Stromal tumor-cell interactions regulate CCL2 expression

Cells do not act in isolation. They coordinate to complete pathological tasks through interactions and the release of cytokines. Some studies have revealed the regulation of CCL2 production by the interaction between tumor cells and stromal cells, but the mechanisms are mostly unknown and deserve further investigation. In hepatocellular carcinoma (HCC), The indirect co-culture system revealed that TAMs released more CCL2 into the culture medium when they were cultured with HCC cells than when they were cultured alone. NF-κB was also enhanced in the co-culture system, which might be related to the upregulation of CCL2 expression (166). In oral squamous cell carcinoma (OSCC), CCL2 release was significantly increased in the coculture system compared with that in the conditioned medium of OSCC cell lines PCI-13 and LUVA (MCs). But the mechanism of this process remains unknown (23). The interaction between epithelial cells and BC cells significantly elevated CCL2 expression levels by up to 8 folds, possibly by influencing mRNA and post-translational modifications (167). In prostate cancer, tumor-derived CXCL8 induced *CCL2* gene expression in WPMY-1 fibroblasts and THP-1 cells (168). TNF-α and IL-1β secreted by stromal cells and BC cells synergistically stimulate mesenchymal stem cells and CAFs to release CCL2 (169). The levels of CCL2 detected in the culture supernatants of cancer-associated adipocytes cocultured with MCF-7 or MDA-MB-231 cells were higher than those in the normal breast adipocytes cocultured with BC cells (170).

## 4 The role of CCL2 in tumors

Cancer cells establish a microenvironment that facilitates tumor progression by recruiting and reprogramming non-cancerous host cells, as well as remodeling the vasculature and ECM. This dynamic process relies on heterotypic interactions between cancer cells and resident or recruited non-cancer cells in the TME (171). CCL2 plays an important role in the survival, proliferation, migration and colonization of distant organs of cancer cells through paracrine and autocrine mechanisms, which runs through all stages of cancer development (172, 173). A large number of pan-cancer analyses have shown that high levels of CCL2 are associated with more aggressive malignancies, higher risk of metastasis, and poor cancer prognosis (174). The high expression of CCL2 may also be used as a potential diagnostic marker for early detection and prognosis evaluation of tumors (175).

### 4.1 CCL2 acts in a paracrine and autocrine manner to stimulate cancer cell proliferation and migration

**Figure 4:**
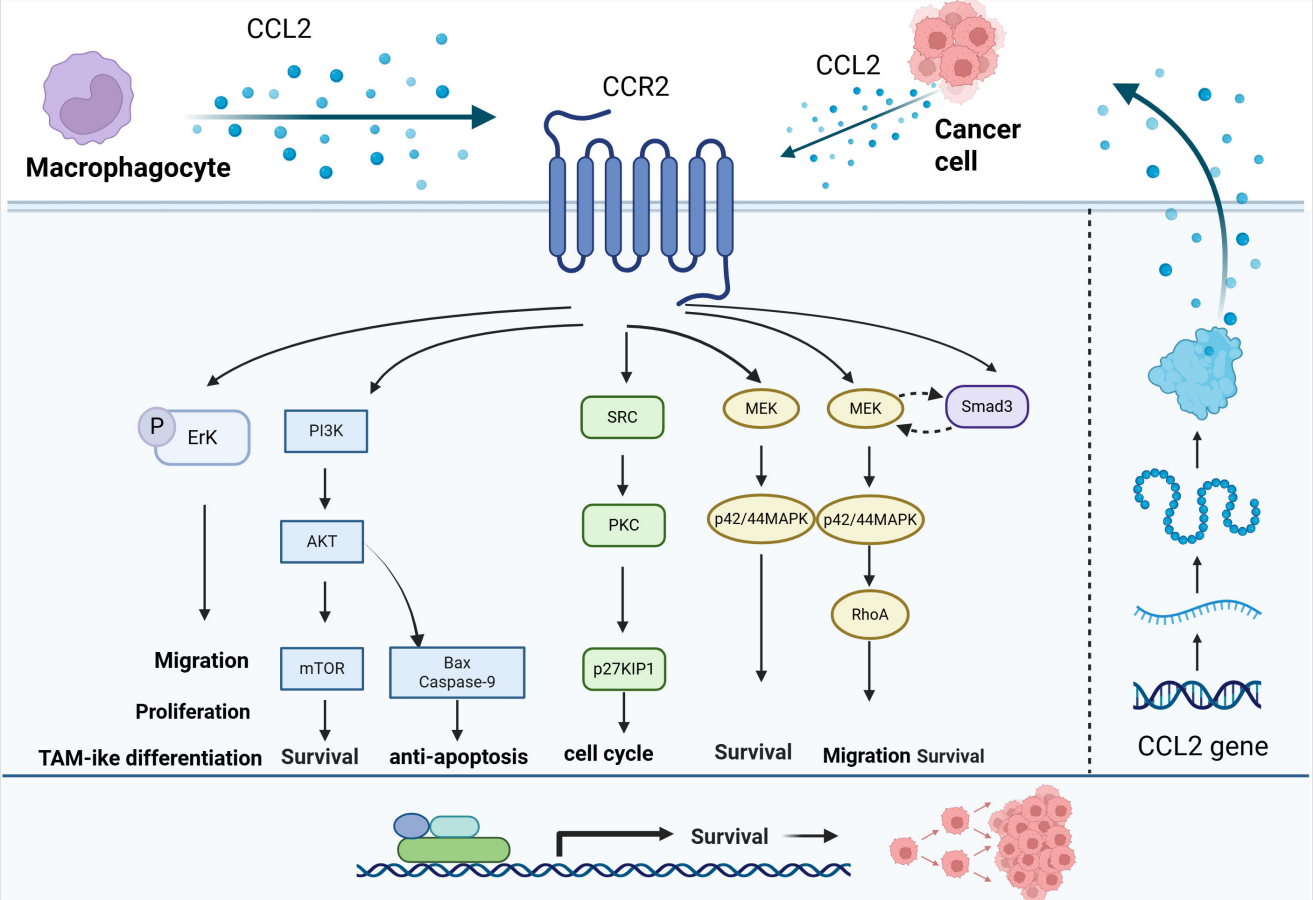
Effect of CCL2 on the proliferation of tumor cells. CCL2 can activate downstream pathways such as PI3K/AKT, SRC/PKC, and MEK-p42/44MAPK through CCR2, up-regulate the expression of anti-apoptotic proteins and cell cycle regulatory proteins, and promote the survival and anti-apoptotic ability of tumor cells.

In the TME, CCL2 secreted by cancer cells and stromal cells can activate downstream pathways, up-regulate the expression of anti-apoptotic proteins and cell cycle regulatory proteins to promote the survival and anti-apoptotic ability of tumor cells (**Figure 4**).

In the indirect co-culture system of HCC and macrophages, CCL2 secretion was observed to be significantly increased, and the proliferation and migration ability of the two types of cells were promoted via autocrine and paracrine mechanisms. The CCL2-CCR2 axis also regulated the phenotypes of HCC cells and macrophages through downstream Erk (176). Through PI3K/AKT, CCL2 can inhibit the expression of pro-apoptotic proteins Bax and Caspase-9, leading to docetaxel (DTX) resistance in lung cancer (177). CCL2 regulates autophagy in a PI3K/AKT-dependent manner through the mTOR pathway and upregulates the expression of Survivin, conferring a significant survival advantage to cells (178). In BC cells, CCL2 binds to CCR2 receptors to activate Smad3 and p42/44MAPK signaling pathways. Specifically, CCL2 induces MEK signaling independently of Smad3 in the p42/44MAPK pathway, thereby promoting cell survival. In addition, CCL2 also regulates cell motility and survival through a RhoA-dependent mechanism by activating Smad3, allowing it to act synergistically with the MEK-p42/44MAPK pathway (179). In BC, CCL2 activates SRC and PKC signaling pathways through its receptor CCR2, which in turn negatively regulates the expression of cell cycle inhibitory protein p27KIP1 and releases cell cycle inhibition (180).

### 4.2 CCL2 directly or indirectly promotes angiogenesis

Angiogenesis, the process of developing new blood vessels, is essential for tumorigenesis. Tumor blood vessels are constantly exposed to factors such as vascular endothelial growth factor (VEGF) and angiopoietin (Ang), leading to vascular system disorder and leakage. This affects tumor oxygenation, alters immune cell kinetics, and reduces drug penetration into tumors, thus promoting high proliferation rates and drug resistance in tumors (181).

**Figure 5:**
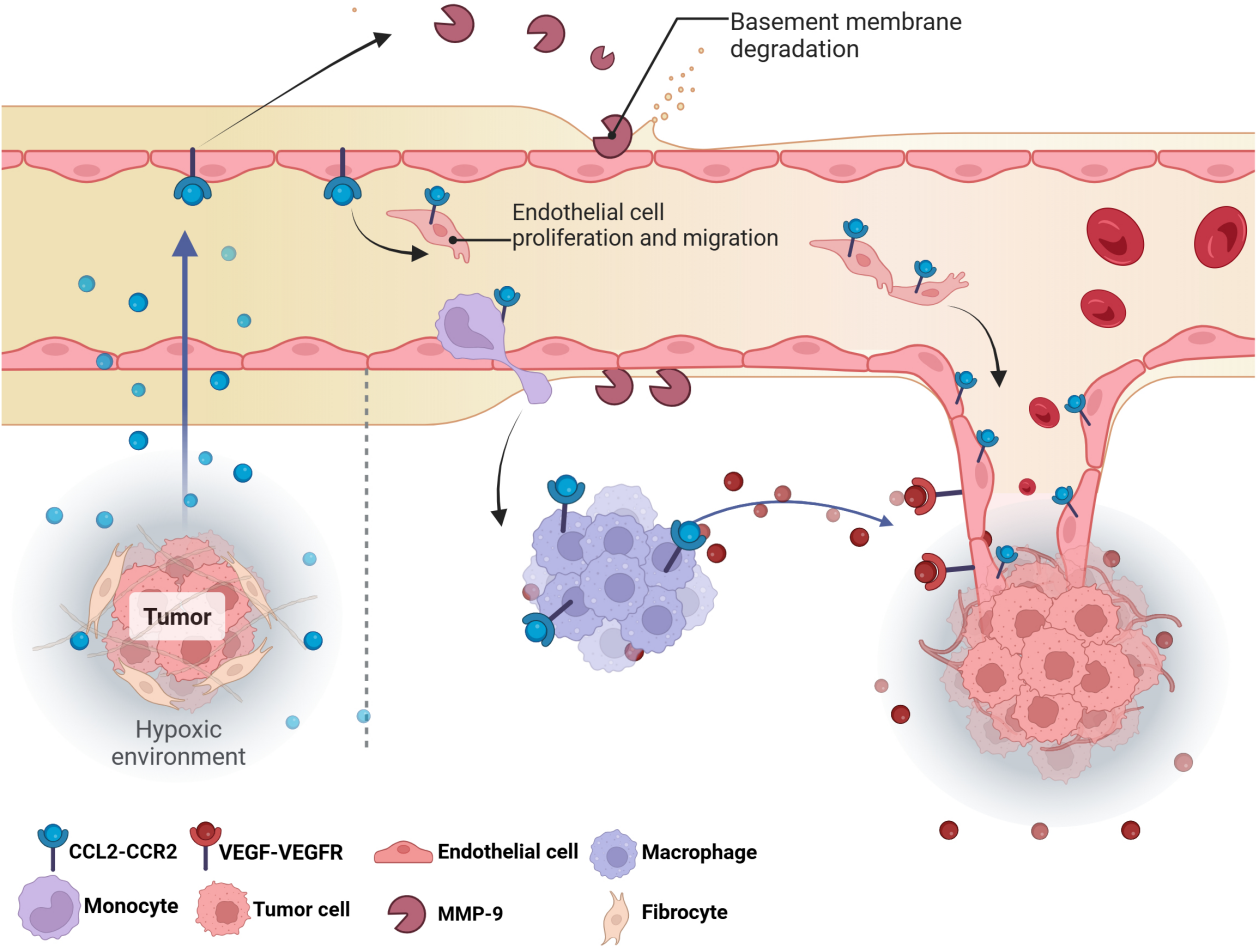
The effect of CCL2 on tumor angiogenesis. The hypoxic environment in tumors triggers the release of CCL2, directly stimulating the proliferation and migration of endothelial cells to form new blood vessels. Meanwhile, macrophages and monocytes are recruited to the tumor site by CCL2, which release MMP-9 and VEGF to degrade the basement membrane and create space for angiogenesis, thereby stimulating angiogenesis.VEGF, Vascular endothelial growth factor; MMP-9; Matrix metalloproteinase-9.

Recent studies have shown that CCL2 forms a complex pro-angiogenic network by directly acting on vascular endothelial cells or indirectly regulating the functions of immune cells and stromal cells (**Figure 5**). *In vitro* experiments, CCL2 can directly promote the tubular structure of human umbilical vein endothelial cells (HUVECs) to form blood vessels without the involvement of inflammatory cells (182). CCL2 also promotes the proliferation and migration of HUVECs through MAPK/ERK1/2/MMP9, PI3K/AKT and Wnt/β-catenin signaling pathways, degrades the ECM and provides space for angiogenesis. At the same time, CCL2 can up-regulate VEGF in endothelial cells to promote angiogenesis (183).

In addition to directly acting on endothelial cells, CCL2 can also act on helper cells in the TME to indirectly regulate angiogenesis. Most of the stromal cells in tumor tissue are fibroblasts. These cells are the main source of CCL2 (184). Additionally, immune cells, especially macrophages and neutrophils, are also key sources of angiogenesis related chemokines, growth factors and proteases (185). CCL2 is a key chemotactic factor for monocytes/macrophages (185). *In vitro* experiments showed that upregulation of Twist1 in cancer cells activated CCL2 mRNA transcription; A chemotactic gradient is generated by the secretion of CCL2 protein to promote macrophage infiltration and subsequent angiogenesis stimulation (160). These TAMs amplify pro-angiogenic signals by secreting VEGF, FGF, IL-8 and other pro-angiogenic factors, releasing MMP-9 to reconstitute the extracellular matrix (186).

Tissue hypoxia is a characteristic of tumor tissue and the main trigger of angiogenesis (107). Many molecules such as VEGF that respond to hypoxia can promote angiogenesis. As mentioned above, both chronic hypoxia and cyclic hypoxia can significantly up-regulate the expression of CCL2, and a large amount of CCL2 will escort the angiogenesis, forming a vicious cycle. Therefore, CCL2 constructs a multi-layered pro-angiogenic network by directly activating endothelial cell signaling pathways and indirectly regulating TAMs and pro-angiogenic factors.

### 4.3 CCL2 is involved in tumor metastasis

Metastasis is the leading cause of cancer-related death in humans (187). Tumor metastasis begins with local invasion. Tumor cells alter their intrinsic characteristics, degrade the extracellular matrix, modulate the immune microenvironment, and acquire invasive capabilities. Subsequently, tumor cells intravasate into blood vessels or lymphatic vessels and inhibit anoikis to survive in the circulation. They then prepare for colonization by forming a premetastatic niche (PMN) in distal organs. Tumor cells then extravasate from blood vessels, proliferate in distant organs, form metastases, and harvest nutrients through angiogenesis. Eventually, micrometastases continue to grow, evade immune attack, and develop into detectable metastases (15). The role of CCL2 in metastasis is pleiotropic, and it is associated with many metastatic steps, such as local invasion, intravasation, PMN formation, anoikis, extravasation, chemotaxis, and colonization (188).

#### 3.3.1 Epithelial-mesenchymal transition (EMT) and local invasion

Once the tumor has successfully formed blood vessels, it can enter the next stage of disease progression, namely local invasion. Aggressive growth is one of the important characteristics of cancer, separating from neighboring cancer cells by breaking the adhesion between epithelial cells and tumor cells, accompanied by an EMT-like state (15).

**Figure 6:**
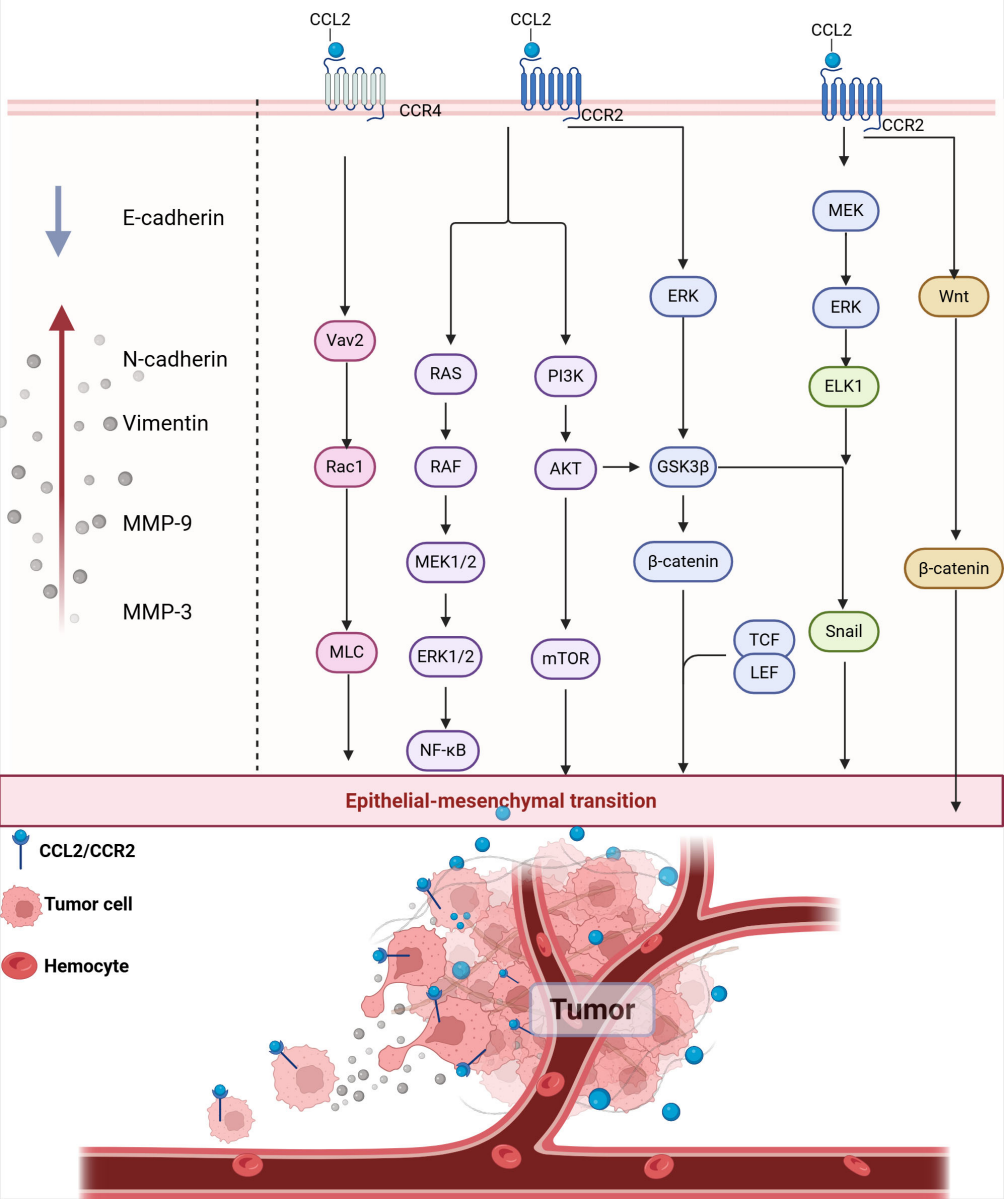
CCL2 promotes EMT and local invasion. CCL2 binding to CCR2 activates multiple signaling pathways, including the RAS/RAF/MEK1/2, PI3K/AKT/mTOR, and ERK pathways. These pathways converge to activate transcription factors such as NF-κB, Snail, TCF/LEF, and β-catenin, which promote the expression of EMT markers. E-cadherin, Epithelial Cadherin; N-cadherin, Neural cadherin; MMP-3, Matrix metallopeptidase 3; MMP-9, Matrix metallopeptidase 9.

CCL2 regulates the expression of EMT markers and promotes the expression and activity of matrix metalloproteinases through downstream signaling pathways. This contributes to increased tumor cell motility and invasiveness, ultimately facilitating local tumor invasion and distant metastasis (**Figure 6**). Recent studies have shown that CCL2 expression can predict clinical outcomes, activate the EMT pathway and angiogenesis in pituitary tumors (189). CCL2 secreted by TAMs can activate the PI3K/Akt signaling pathway, increase the expression of β-catenin and bind to TCF/LEF transcription factors, resulting in the down-regulation of epithelial markers E-cadherin and up-regulation of interstitial markers such as N-cadherin and Vimentin, which endow cancer cells with the ability of migration and invasion (190). CCL2 also induces ERK/GSK-3β/Snail signaling to promote EMT and the migration of MCF-7 human breast cancer cells (191). M2-like TAMs activate the MEK/ERK/ELK1/Snail signaling pathway in gallbladder cancer (GBC) cells by secreting CCL2, thereby promoting EMT, stemness and metastasis of GBC (192). *In vitro* experiments, CCL2-CCR2 axis was found to induce invasion and EMT of hepatocellular carcinoma *in vitro* by activating Hedgehog signaling pathway (193). In the pre-metastatic microenvironment, CCL2 secreted by cancer cells and bone marrow cells attracts and induces M2-TAM and other cells to activate Wnt/β-catenin signaling pathway, which destroys the E-cadherin junction between early cancer cells. Prior to tumor formation, these processes facilitate the establishment of an early disseminated microenvironment, enhance the active invagination of tumor cells and their entry into the circulatory system, and ultimately facilitate metastasis to distant organs (194). In addition, pharmacological inhibition of CCL2/CCR4/Vav2/Rac1/MLC signaling pathway can effectively reduce the motility of cancer cells, thereby inhibiting local invasion and distant lymph node metastasis in head and neck squamous cell carcinoma (HNSCC) cells (33). These results suggest that CCL2-CCR4 may serve as a potential therapeutic molecular target by inhibiting tumor invasion and metastasis.

Matrix metalloproteinase (MMP) can degrade the extracellular matrix and basement membrane through its proteolytic activity, make cancer cells transition from epithelial to mesenchymal state, and enhance cell motility and invasion ability. MMP also creates conditions for tumor cells to enter blood vessels or lymphatic vessels by destroying the vascular endothelial cells layer (195). CCL2-CCR2 axis can induce EMT by promoting the recruitment of MMP in the extracellular matrix, which leads to the degradation of tight junction proteins and basement membranes. In non-small cell lung cancer (NSCLC), CCL2 reduces E-cadherin levels and upregulates vimentin, MMP-2 and MMP-9 protein levels through PI3K/Akt/mTOR to activate EMT (196). In GBM induced by the mitochondrial pro-apoptotic protein BV6, NF-κB activation induces the upregulation of CCL2 and triggers the expression of MMP-9 in an autocrine/paracrine manner, promoting the migration and invasion of GBM cells (197). CCL2 also increases the level of MMP-9 in human chondrosarcoma cells via the Ras/Raf/MEK/ERK/NF-κB signaling pathway to promote cell motility (198). *In vivo* and *in vitro* experiments with pulmonary metastatic osteosarcoma showed that CCL2 enhances MMP-3 dependent cell movement by inhibiting miR-3659 synthesis (199).

#### 3.3.2 Immunosuppression and Pre-metastatic Niche formation

The PMN refers to a specialized microenvironment that facilitates the colonization and growth of tumor cells in the distal organ. This is achieved through the secretion of factors and extracellular vesicles by the primary tumor prior to the arrival of tumor cells at the distal site. This concept was first proposed by Kaplan et al., in 2005 (200).

**Figure 7:**
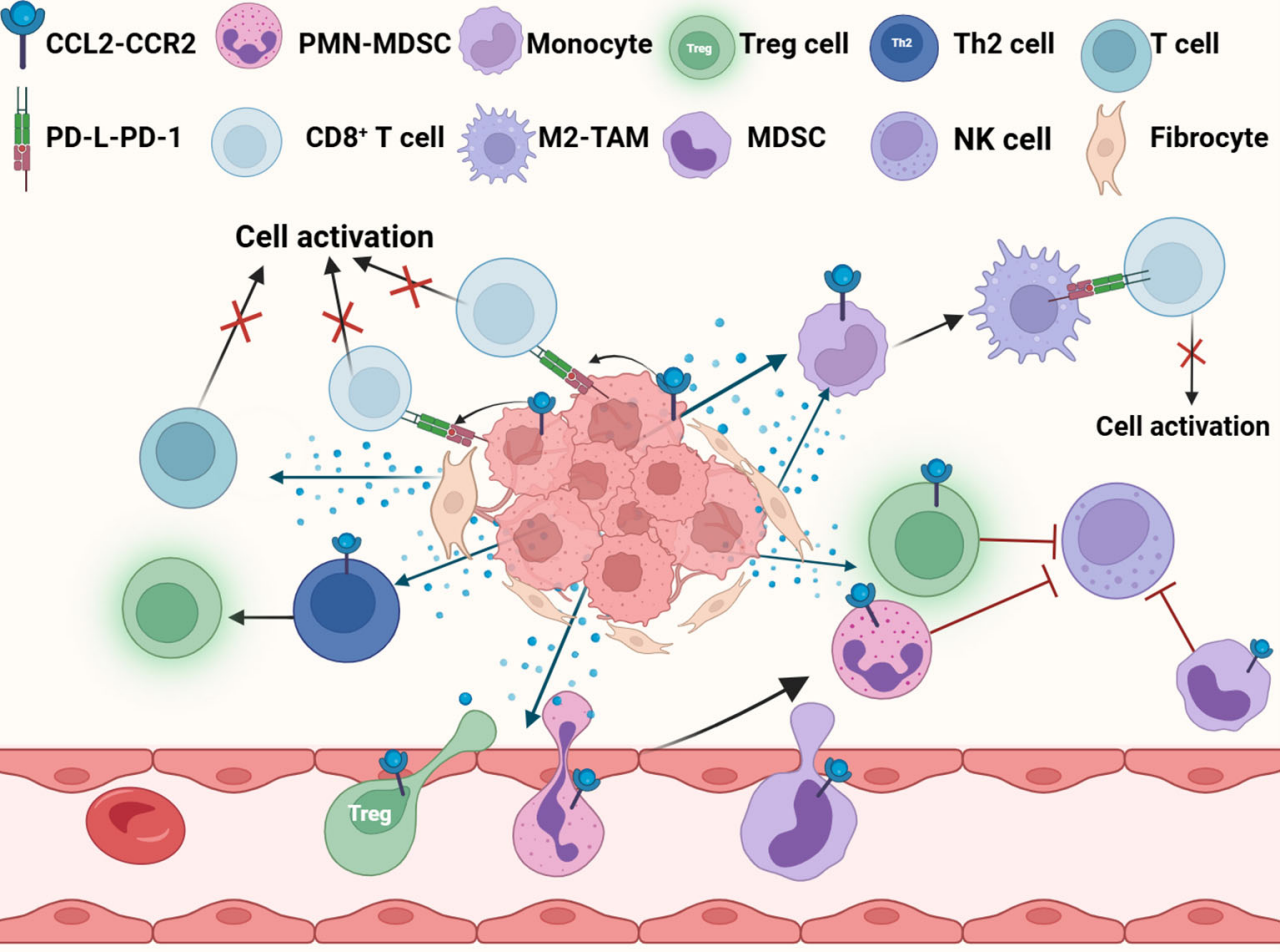
CCL2 promotes immunosuppression. CCL2 fosters the establishment of an immunosuppressive microenvironment by recruiting immunosuppressive cells, suppressing immune cell activity, and upregulating PD-L1 expression in tumor cells. M2-TAM, M2-tumor associated macrophage; Treg cell, Regulatory T cell; MDSC, Myeloid-derived suppressor cell; PMN-MDSC, Polymorphonuclear myeloid-derived suppressor Cells; NK cell, Natural killer cell.

CCL2 provides support for tumor cell metastasis by not only activating and recruiting immunosuppressive cells, but also by directly inhibiting immune cell activity, upregulate the expression of immune checkpoints, acting together on the formation of the PMN (**Figure 7**). As a regulator of T cells, CCL2 regulates the differentiation of T helper 2 cells (Th2 cells) into more immunosuppressive regulatory T cells (Treg cells) (201). CCL2 can also recruit bone MDSCs and regulatory Treg cells through CCR2 and CCR4 receptors, and the two synergistically inhibit the clearance of glioma cells by cytotoxic T cells (202). In the lung metastasis model of renal cell carcinoma, CCL2 secreted by lung macrophages can attract TAM and polymorphonuclear myeloid-derived suppressor cells (PMN-MDSC), and the aggregation of these immunosuppressive cells forms an immunosuppressive and metastatic microenvironment (203). Under hypoxic conditions, activation of the lactate receptor HCAR1 signaling pathway induces the expression of CCL2 and CCL7 in colorectal tumor cells, which then attracts immunosuppressive CCR2^+^ PMN-MDSCs into the TME (117). CCL2 also activates the STAT3 signaling pathway by binding to the receptors on the surface of PMN-MDSCs, thereby regulating the expression of calcium-binding proteins gp91phox, S100A8 and S100A9, enhancing the production of ROS and inhibiting the ability of T cells (204). Furthermore, CCL2 can also suppress cytotoxic NK cells in the pre-metastatic niche, helping triple-negative breast cancer cells evade immune surveillance and thereby promoting their metastasis (205). In gastric cancer, CCL2 ^+^ fibroblasts activate M2-TAM via STAT3 while inhibiting T cell activation and also inducing the production of inhibitory TME (206).

Additionally, CCL2 induces the upregulation of programmed death ligand 1 (PD-L1) and programmed death ligand 2 (PD-L2) in tumor cells and immune cells, and inhibit the activation of CD8+ T cells mediated by PD-1 antibodies. CCl2-induced M2 polarization increases the expression of PD-L1 and PD-L2 in TAMs, which transmit inhibitory signals to CD8 ^+^ T cells through PD-1 signaling and helps tumor cells escape the attack of the immune system (207, 208). Even when PD-L1 is completely blocked, CCL2 release can still counteract the effect of PD-1/PD-L1 inhibitors by recruiting immunosuppressive cells into the TME (209).

#### 3.3.3 Tumor cell extravasation

**Figure 8:**
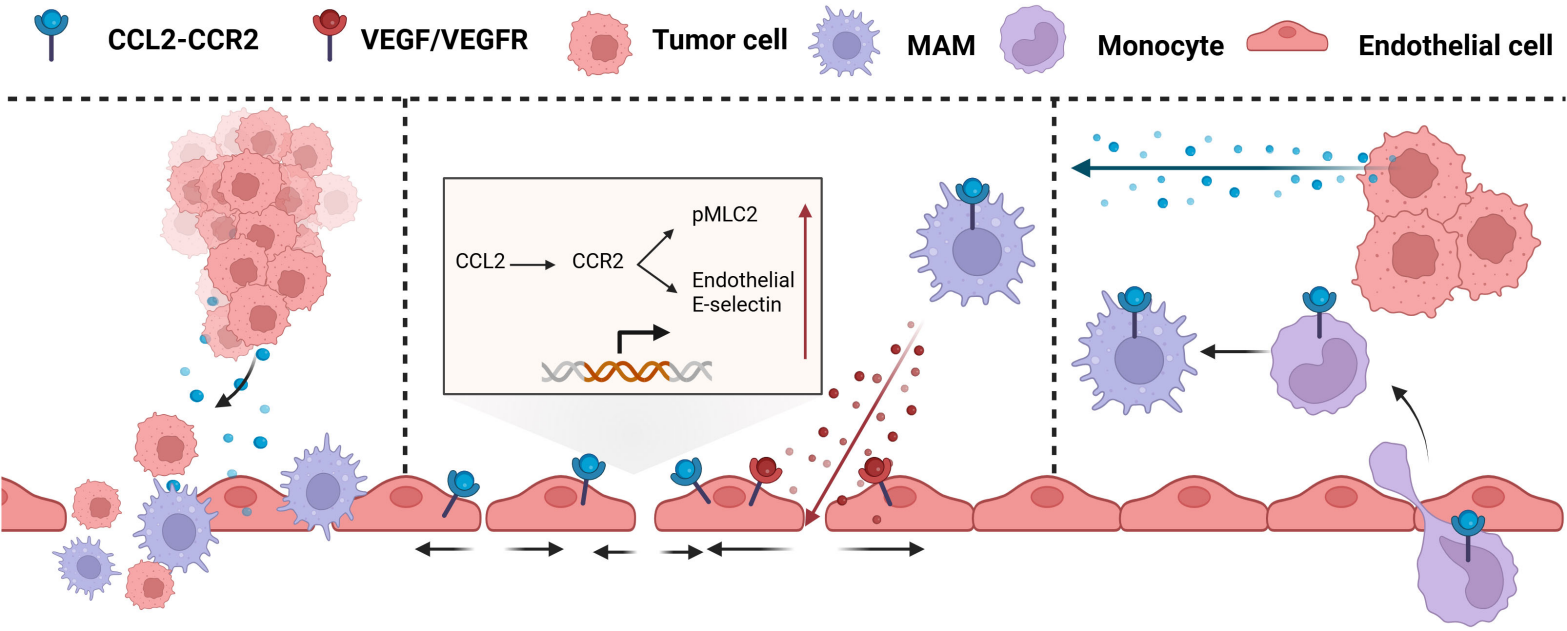
CCL2 promotes tumor extravasation. CCL2 chemotactic CCR2+ Ly6C high expression monocytes act as carriers through the vascular wall and enhance local vascular permeability through endothelial cells. MAM, Mammalian Target of Rapamycin; VEGF, Vascular Endothelial growth factor; VEGFR, Vascular endothelial growth factor receptor.

Tumor Cell Extravasation refers to the process by which circulating tumor cells (CTCs) pass through the endothelial cell layer from blood vessels or lymphatic vessels and enter distant organs or tissues (210). CCL2 secreted by tumor cells promotes cancer cell extravasation through a dual mechanism (**Figure 8**) (1): CCL2 attracts CCR2^+^ Ly6C high expressing monocytes, which are used by tumor cells as carriers across the vessel wall (2); CCL2 enhances the permeability of local blood vessels through endothelial cells (210).

At the site of metastasis, inflammatory monocytes, their derivatives, and metastasis-associated macrophages (MAMs) are critical in facilitating the extravasation and proliferation of metastatic tumor cells. Inflammatory monocytes, which highly express CCR2 and respond to CCL2 produced by tumor and stromal cells, are recruited to metastatic sites such as the lungs and liver before tumor cells and differentiate into MAMs, secreting VEGF and promoting tumor cell metastasis (211, 212). The CCL2-induced CCL3 cascade also prolongs the retention time of MAMs at the tumor site, thereby accelerating the extravasation of tumor cells (213). However, it has been demonstrated in mouse models of colon cancer lung metastasis that CCL2-induced high expression of Ly6C monocytes is a necessary but not sufficient condition for effective metastasis. The loss of CCR2 in monocytes reduces but does not prevent metastasis, while the absence of CCR2 in endothelial cells prevents lung metastasis (214).

Endothelial retraction is a key step for tumors to undergo intravasation and extravasation, which promotes tumor metastasis by enhancing the contraction of vascular endothelial cells and intercellular space. In the lung metastasis model, CCL2 stimulates pulmonary endothelial cells, leading to the phosphorylation of myosin light chain (MLC2) and the upregulation of E-selectin expression on endothelial cells. This process triggers cytoskeletal rearrangement and facilitates chemotactic recruitment, adhesion, and activation of monocytes, thereby shaping the TME. Additionally, it enhances vascular permeability, which is essential for initiating metastasis, and promotes the tranendothelial migration of tumor cells (215). Furthermore, CCL2 derived from tumor cells can activate the CCR2 receptor on vascular endothelial cells, inducing the JAK2/STAT5 and p38MAPK signaling pathways. The two pathways act in concert to increase (214).

In conclusion, CCL2 acts as a core driver of tumor metastasis by regulating EMT, ECM degradation, vascular leakage, and premetastatic niche formation. CCL2 plays a crucial role throughout the entire process of metastasis, including initiation, progression, and colonization, forming a complex interaction network with immune cells and stromal cells within the TME.

## 5 The clinical efficacy and potential breakthrough areas of CCL2

Previous literature has systematically described the current status of clinical treatment of CCL2 in tumors (18, 216). this article will focus on the clinical efficacy and extension of targeting CCL2.

### 5.1 The clinical efficacy and limitations of CCL2

Immunomodulatory antibodies and small molecule inhibitors targeting cancer cells have shown definite efficacy in a variety of solid tumors such as breast cancer and lung cancer (217). Among them, neutralizing antibodies are still the main means to interfere with CCL2/CCR2 signaling axis. In theory, CCL2 blockade can simultaneously inhibit the recruitment of immunosuppressive cells and enhance the infiltration of killer T cells to achieve tumor suppression, but the clinical benefit of targeting the CCL2/CCR2 axis is still pending.

In the case of S0916 (MLN1202; prozolizumab; TAK-202), a humanized IgG1 monoclonal antibody targeting CCR2, the results of a phase II study (NCT01015560) in bone metastases or solid tumors have not been published, and its anticancer activity or potential toxicity cannot be confirmed at this time (218). Another phase 1 study in advanced melanoma (NCT02723006) was stopped early because of a 58% incidence of serious adverse events (219). In contrast, the CCR2 inhibitor CCX872 showed benefit over single-agent chemotherapy in combination with FOLFIRINOX in a phase Ib trial of pancreatic cancer (220).

Carlumab (CNTO888), the first human recombinant monoclonal antibody (IgG1κ) to enter the clinic, significantly reduced the infiltration of tumor-associated macrophages and inhibited tumor growth and metastasis in preclinical models. However, the clinical efficacy of Carlumab is far from expected. In a phase I trial in patients with advanced solid tumors (NCT00537368), free CCL2 was only transiently inhibited after the first dose, followed by a rapid rebound and above baseline levels (221); Total CCL2 (including complexes) can increase up to thousfold, and no clear clinical efficacy has been observed (221). In another phase I study (NCT01204996), Carlumab combined with chemotherapy failed to enhance the efficacy of chemotherapy and did not prolong the inhibition of free CCL2 (222). In a phase II trial in metastatic castration-resistant prostate cancer (NCT00992186), free CCL2 showed a trend of rapid rebound despite a manageable safety profile (223). Pharmacokinetic analysis further revealed that Carlumab could not achieve sustained CCL2 neutralization due to its large fluctuation in blood concentration and short half-life (about 2.4 days) (224). In addition, studies in breast cancer models have shown that withdrawal of CCL2 inhibitors triggers massive monocyte mobilization, cancer cell activation, and IL-6/VEGF-A-driven metastatic recurrence, whereas concurrent blockade of CCL2 and IL-6 significantly inhibits metastasis and extends survival (225). In summary, the reasons why CCL2 neutralizing antibodies have been frustrated in clinical trials can be summarized as follows:

1. Poor pharmacokinetics and rebound effect: the antibody has insufficient affinity with CCL2 and the complex is easy to dissociate; At the same time, the body’s synthesis rate of CCL2 is extremely fast, which together lead to the unstable maintenance of blood drug concentration, and the level of CCL2 often rebound sharply after drug administration.
2. Limitations of the mechanism of action: Neutralization strategies can only temporarily block extracellular CCL2 but cannot silence CCL2 at its source, thus failing to restore the homeostasis of CCL2 in the tumor microenvironment.
3. Signaling compensatory network: A single CCL2 blockade triggers a wide range of compensatory mechanisms. This involves not only the compensatory upregulation of CCL2, but is frequently accompanied by increased levels of other key inflammatory factors, such as IL-6 and TNF-α, leading to the establishment of one or more alternative inflammatory pathways that bypass the initial therapeutic targets.

This is the common problem of all neutralizing antibody therapy, and it is also the breakthrough point of precision medicine.

### 5.2 Future development direction

Although the current strategies to target CCL2 face challenges, emerging studies are attempting from the aspects of source intervention and synergistic blockade, which provide new directions for overcoming the existing limitations.

#### 5.2.1 Interfering with CCL2/CCR2 signaling axis at the source

Source inhibition at the level of gene expression is a feasible strategy to achieve long-term blockade. For example, the Ca-TAT nanocomplexes are formed through non-covalent cross-linking mediated by calcium ions, which enable the complexation of siRNA with TAT cell-penetrating peptides. Topical delivery of CCL2 siRNA via Ca-TAT nanocomplexes can achieve efficient and durable gene silencing in tumor cells with minimal effect on normal tissues. Similarly, siRNA delivery using polyethylenimide-coated mesoporous silica nanoparticles effectively silenced TWIST1 and inhibited CCL2 expression, thereby inhibiting tumor growth in an *in vivo* model. In addition to nucleic acid drugs, small molecule drugs such as Bindarit can reduce the synthesis of CCL2 by inhibiting AKT and NF-κB pathways, showing potential inhibitory effects on tumor progression and metastasis (226). The sicCR2-encapsulated nanoparticles (CNP/siCCR2) can effectively silence the CCR2 expression of macrophages, inhibit the secretion of IL-10 and TGF-β immunosuppressive factors, and improve the cytotoxic efficacy of adriamycin. It can significantly inhibit the function of CCL2 and modify the immunosuppressive tumor microenvironment (227). In addition, targeted regulation of upstream signaling nodes is also an important direction. TNF-α inhibitors such as etanercept and adalimumab can inhibit the expression of CCL2 in monocytes through MAPK, NF-κB and epigenetic pathways. microRNA-206 can inhibit the induced expression of CCL2 in tumor-associated macrophages and cytotoxic T lymphocytes by regulating the Kruppel-like factor 4 (Klf4) transcription factor.

#### 5.2.2 Synergistic blocking and combined treatment strategies

In view of the redundancy and compensatory mechanism of CCL2 signaling network, combined targeting of its cofactors has become the key to improve the therapeutic effect. In the pancreatic cancer model, although inhibition of CCR2 alone can reduce TAM infiltration, it leads to a compensatory accumulation of CXCR2^+^ TAN. Meanwhile, simultaneous blockade of CCR2 and CXCR2 can synergistically inhibit tumor and enhance chemotherapy response (228). The synergistic axis formed by IL-6 and CCL2 is of particular interest: both of them are often up-regulated functionally by p53 isoforms to promote metastasis (103). Compound N-2 could inhibit tumor growth by synergistically inhibiting the p53 and NF-κB pathways and simultaneously reducing the levels of IL-6 and CCL2 (104). CMTM4 could also induce both CCL2 and IL-6 to promote the expansion of MDSCs, while its knockdown enhanced the anti-PD-1 efficacy (106). In recurrent head and neck squamous cell carcinoma, combined blockade of IL-6 and CCR2 can significantly enhance NK cell activity, and the effect is better than that of single pathway inhibition (229), suggesting that this combined strategy has the potential to reverse the mask transformation of immunotherapy resistance.

In addition, CCL2 also synergetic with HGF/MET pathway to promote the progression and metabolic reprogramming of breast cancer, and the combination of CCR2 knockout and MET inhibitor meritinib can more effectively inhibit tumor growth and stromal response (230). At the same time, CCL2 is also involved in the resistance mechanism of PD-1/PD-L1 inhibitors: when PI3K/AKT and NF-κB pathways jointly activate CCL2 and PD-L1, even if PD-L1 is blocked, the release of CCL2 may still lead to treatment failure. Dual inhibition of PD-L1 and CCL2 could reverse the drug resistance phenotype. On the basis of this, a phase I clinical trial (NCT02723006) evaluating the combination of CCR2 inhibitors and nivolumab in the treatment of advanced melanoma is ongoing to verify the synergistic effect of combined blockade of CCR2 and immune checkpoints in activating CD8^+^ T cells and overcoming immunosuppression (209).

It must be pointed out that the majority of the aforementioned therapies are still in the preclinical research stage. Section 2 of this article attempts to systematically explain the upstream regulatory mechanisms of CCL2 in tumors, from gene transcription, mRNA regulation to protein expression and secretion, and even the complex downstream synergistic effects. For this reason, each link in this pathway constitutes a potential intervention target. Future research should avoid the limitations of single therapy: first, to deeply analyze the upstream generation mechanism of CCL2 to develop more fundamental intervention methods; second, to clarify its downstream compensatory and synergistic networks, laying a theoretical foundation for the design of effective combination therapies.

## 6 Conclusions and future perspectives

This work systematically reviews the production mechanism of CCL2 in a variety of tumors and its multifaceted role in the TME, focusing on its upstream regulation and downstream effects. The aim is to comprehensively clarify the production, regulation, and key biological processes of CCL2 in the TME and provide theoretical basis and potential targets for the development of CCL2 targeted therapy.

CCL2 is continuously produced by tumor cells and stromal cells through three main pathways: constitutive secretion, microenvironmental stimulation response and tumor-stroma interaction. Among them, SNPs, epigenetic regulation and noncoding RNA regulation are the core mechanisms of CCL2 constitutive expression. External inflammatory factors in the TME (such as TNF-α, IL-1β, and IL-6) and hypoxia signals further amplify the secretion of CCL2 by activating NF-κB and other transcription factor pathways. This continuous release of CCL2 reshapes the immune microenvironment by recruiting TAMs and MDSCs and forms a positive feedback loop through autocrine and paracrine signals. These interesting crosstalks are the reasons for breaking CCL2 homeostasis and should be considered for future treatments.

As a “good soldier” of tumor cells, the activation of the CCL2–CCR2 axis directly induces the expression of EMT-related transcription factors and enhances the invasion ability of cancer cells by regulating PI3K/Akt, MAPK, NF-κB, and other signaling pathways. CCL2 also promotes the secretion of MMPs and the release of VEGF, accelerates the degradation of ECM and angiogenesis, and creates conditions for the intravasation of tumor cells and distal colonization of CTCs. In PMN formation, CCL2 recruits immunosuppressive cells and inhibits the activity of NK and other immune cells, providing a “protective umbrella” for the immune escape of metastases.

To date, the downstream effects mediated by CCL2 have been clearly elucidated. In clinical intervention strategies, small-molecule inhibitors and neutralizing antibodies are mainly used to block the CCL2–CCR2 signaling axis (221, 231). However, these monotherapies have not significantly improved the prognosis of patients. In preclinical studies and clinical trials, the use of CNTO888 against CCL2 or MLN1202 antihuman antibodies against CCR2 in different cancer types have only achieved short-term and hardly predictable anticancer responses (18). These negative outcomes can be attributed to its complex upstream regulatory network and compensatory mechanisms.

Recent studies have shown that targeting the upstream signals of CCL2 modified by lactic acid and the STAT3 axis overcomes the immunotherapy resistance of pancreatic ductal adenocarcinoma (144). microRNA-206 regulates CCL2 expression through mRNA and promotes the recruitment of cytotoxic T lymphocytes (CTLS) via CCR2 in the liver. Targeting microRNA-206 inhibits its CCL2 induction in macrophages (71). Moreover, the combination of CCL2 inhibition with an immunomodulator targeting PD-1/PD-L1 has shown more favorable results than each monotherapy (32). These studies suggest that silencing CCL2 at its source (such as mRNA interference, siRNA, epigenetic modulation, or promoter inhibition), and combined with immunomodulatory agents to disrupt its positive feedback loop with PD-L1, TNF-α, IL-1β, and IL-6, may constitute a potentially effective cancer treatment strategy.

In summary, the mechanism of CCL2 in the TME is complex and multifaceted. Therapeutic strategies targeting CCL2 still require a deep understanding of its upstream and downstream mechanisms in tumors, and effective intervention can be achieved only by fully grasping the complexity of its function. The development of combined strategies or source regulation of CCL2 may be the direction of future research.

## Glossary

AP-1: Activator protein 1
Ang: Angiopoietin
BC: Breast cancer
BLCA: Bladder cancer
CCR2: C-C chemokine receptor type 2
CAF: Cancer-associated fibroblasts
CBP: Creb-binding protein
CCL2: Chemokine ligand 2
CRC: Colorectal cancer
CTCs: Circulating tumor cells
CNP/siCCR2: sicCR2-encapsulated nanoparticles
DADS: Diallyl disulfide
DNMT1: DNA methyltransferase 1
DTX: Docetaxel
EMT: Epithelial-mesenchymal transition
ECL2: The second extracellular loop
ESCC: Esophageal squamous cell carcinoma
FAP: Fibrocell-activating protein
FoxQ1: Forkhead box Q1
GBC: Gallbladder cancer
HA: Hemangioma
HCC: Hepatocellular carcinoma
HDAC1: Histone deacetylase 1
HDAC5: Histone deacetylase 5
hFOB: Human fetal osteoblasts
HBMECs: Human bone marrow endothelial cells
HUVECs: Human umbilical vein endothelial cells
HNSCC: Head and neck squamous cell carcinoma
HRE: Hypoxia response element
Il-1β: Interleukin-1β
IL-6: Interleukin-6
JHDM: Jumonji histone demethylase
KS: Kaposi’s sarcoma
LC: Lung cancer
LUAD: Lung adenocarcinoma
LncRNA: Long non-coding RNA
LPS: Lipopolysaccharide
MAPK: Mitogen-activated protein kinase
MAMs: Metastasis-associated macrophages
MCP-1: Monocyte chemoattractant protein-1
MDSCs: Myeloid-derived suppressor cells
MCs: Mast cells
MLC2: Myosin light chain
MMP: Matrix metalloproteinase
NBAs: Normal breast adipocytes
NF-κB: Nuclear factor kappa-B
NK: Natural killer
NSCLC: Non-small cell lung cancer
OSCC: Oral squamous cell carcinoma
PDAC: Pancreatic ductal adenocarcinoma
PD-L1: Programmed death ligand 1
PD-L2: Programmed death ligand 2
PMN: Premetastatic niche
PMN-MDSC: Polymorphonuclear myeloid-derived suppressor cells
PTHrP: Parathyroid hormone-related protein
RAGE: Receptor for advanced glycation end
Rgs2: Regulator of G protein signaling 2
SNPs: Single nucleotide polymorphisms
Sp1: Specific Sequence Protein 1
STAT: Signal transducer and activator of transcription
TAM: Tumor-associated macrophages
TME: Tumor microenvironment
TNF-α: Tumor necrosis factor-α
TM3: Third transmembrane domain
Treg cells: T cells
VEGF: Vascular endothelial growth factor
VSMCs: Vascular smooth muscle cells
3’UTR: 3’
